# On-chip solitons are gaining new colors

**DOI:** 10.1038/s41377-026-02313-6

**Published:** 2026-05-08

**Authors:** Grégory Moille, Kartik Srinivasan, Yanne K. Chembo

**Affiliations:** 1 Joint Quantum Institute, NIST/University of Maryland, College Park, MD USA; 2 Microsystems and Nanotechnology Division, National Institute of Standards and Technology, Gaithersburg, MD USA; 3 A. James Clark School of Engineering, Department of Electrical and Computer Engineering, University of Maryland, College Park, MD USA; 4 Institute for Research in Electronics and Applied Physics (IREAP), University of Maryland, College Park, MD USA

**Keywords:** Solitons, Frequency combs

## Abstract

The first experimental demonstration of single-pump multicolor solitons using a triple-microring configuration has been reported recently. This original approach expands the potential of optical frequency comb technology for photonics and time-frequency metrology.

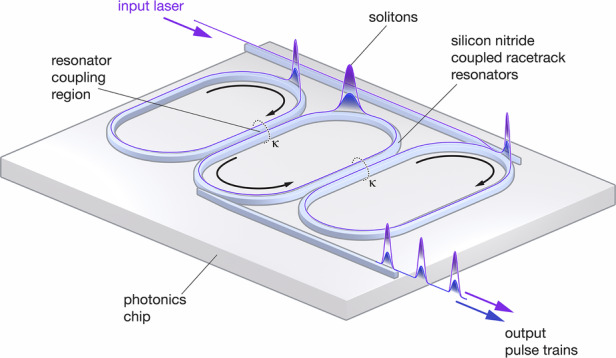

Chip-scale optical frequency combs promise to bring precision measurement and spectroscopy capabilities from laboratory optical tables into portable, integrated devices. Yet realizing this vision confronts a fundamental trade-off in microresonators that generate dissipative Kerr soliton (DKS) frequency combs: small resonators generate broadband combs spanning hundreds of nanometers, ideal for complete comb stabilization^1,2^, but at terahertz repetition rates (the spacing between the comb teeth), too fast for direct electronic detection. Large resonators yield tens of gigahertz repetition rates compatible with standard electronics, and ideal for spectroscopy^3^ and low-noise microwave generation^4,5^, but produce severely bandwidth-limited combs. The culprit is the single anomalous group velocity dispersion (GVD) window that supports conventional DKS formation^6^. Even when dispersive waves extend the spectrum into the normal dispersion regime, the characteristic hyperbolic secant square spectral envelope – determined by the anomalous quadratic dispersion at the pump – decays rapidly, with comb tooth power dropping by tens of decibels for comb teeth far from the pump.

About ten years ago, a theoretical proposal offered a solution: engineer the resonator dispersion with an oscillatory frequency dependence to create multiple anomalous GVD windows^7^. In such a design, the primary soliton would radiate into these additional windows, forming a phase-locked multicolor soliton that extends the comb bandwidth far beyond the single-window limit.

Early proposals to realize this oscillatory dispersion relied on linear coupling between different mode families within a single ring, requiring departure from standard rectangular waveguide cross-sections. Such designs demanded three-dimensional fabrication architectures – either vertically stacked materials^8^ or non-rectangular waveguide geometries^9^ – yet yielded only one additional anomalous dispersion window and remain to be demonstrated to this day on chip. Importantly, multicolor solitons have been demonstrated in table-top fiber systems^10^, where programmable pulse shapers provide the fine dispersion control necessary for experimental realization. This success even further motivates the search for an on-chip implementation.

Driving the microresonator with multiple pump lasers takes a different approach, relying on purely nonlinear effects instead of geometric dispersion engineering: an auxiliary laser nonlinearly mixes with the primary DKS to generate spectral components at new frequencies^11–13^. In the low-dispersion regime typical of on-chip systems, cross-phase modulation naturally locks the group velocities of the different spectral components, binding them temporally^14,15^. In the asynchronous operating regime (where the two pumps maintain independent phase evolution), this technique creates an idler wave from optical parametric oscillation (OPO) from the soliton and auxiliary pump, expanding the comb span over 1.6 octaves^13^.

However, because multi-pumping operates within a single anomalous dispersion window rather than creating new ones, it produces quasi-continuous-wave spectral features with Lorentzian lineshapes, not the bright soliton pulses with sech profiles that would arise from genuine multicolor solitons.

Nevertheless, multi-pumping reveals an important insight into the role of phase synchronization. Asynchronous operation extends the spectrum by generating new frequencies through four-wave mixing; these frequencies are inaccessible when the pumps are phase-locked^16–18^. This mechanism becomes apparent when the dynamics are decomposed into multiple nonlinear coupled wave equations^19^ where a new idler wave, only parametrically driven, must be accounted for. This finding suggests that multicolor solitons may not require full phase synchronization after all, as distinct phase velocities could be essential for generating new frequencies. Yet, multi-pumping neither creates the additional anomalous GVD windows needed to support separate soliton pulses nor scales to the large number of distinct center frequencies envisioned in the original multicolor soliton concept.

In this issue, Ji et al.^20^ report the first demonstration of single-pump multicolor solitons in an integrated microresonator, circumventing the fabrication challenges that stymied earlier geometric designs. The breakthrough builds on the coupled-ring platform’s versatility, demonstrated in recent work by the same group^21,22^, including Yuan et al.‘s demonstration that interband coupling can compensate for normal material dispersion, enabling soliton pulse pairs in resonators where bright solitons would otherwise be forbidden. Although concentric resonators have been shown to engineer group velocity through mode coupling^23,24^, the authors’ three-coupled-ring configuration offers post-fabrication reconfigurability through differential heating.

The key insight: rather than engineering multiple GVD windows within a single mode family, coupled microring resonators create distinct dispersion bands through mode hybridization, as shown in Fig. 1. This configuration enables a different formation mechanism for multicolor solitons. Instead of relying on Cherenkov radiation to bridge spectral gaps, the phase offset between hybridized bands generates an OPO that couples energy between the bands nonlinearly. The OPO signal and idler seed soliton formation in secondary anomalous dispersion windows, achieving the multicolor soliton effect with a single pump, unlike multi-pumping approaches that require separate lasers for each spectral region.

**Fig. 1:**
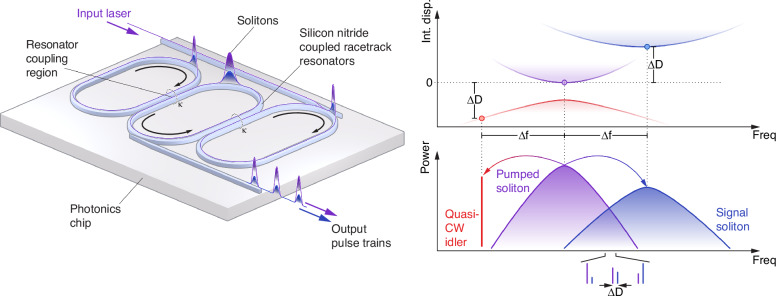
Routes to multicolor solitons. The Ji et al. breakthrough: three coupled rings create distinct dispersion bands linked by optical parametric oscillation. Differential heater tuning enables post-fabrication control of the frequency separation, leading to the generation of chip-scale multicolor solitons

The experimental demonstration confirms this mechanism. Ji et al. observe two temporally bound pulses with durations of 688 fs (primary) and 434 fs (secondary), sharing a repetition rate of approximately 19.86 GHz. The frequency separation between the soliton colors can be electrically tuned from 0.5 THz to 1.5 THz using differential heaters on the coupled rings, a flexibility impossible with fixed geometric designs. As in multi-pumping, cross-phase modulation locks the two solitons to a common group velocity, yet they are not synchronized, maintaining distinct phase velocities that enable OPO formation. This results in two soliton microcombs with a shift in carrier-envelope offset frequencies that can be phase-locked using active stabilization. This multicolor soliton demonstration represents a culmination of this platform development: post-fabrication reconfigurability replaces the rigid geometric engineering that limited earlier proposals.

Several directions emerge from this breakthrough. The tunable terahertz-scale frequency separation between soliton colors opens applications in THz spectroscopy, where the optical beat frequency generates THz-band combs with microwave repetition rates. The system’s reliance on a Moiré effect between the different ring-free spectral ranges creates a periodic GVD profile with multiple anomalous dispersion windows^22^. This periodicity solves the scaling problem that plagued multi-pumping, as a single pump can now generate numerous soliton colors across the periodic dispersion landscape, potentially accessing wavelengths far beyond the reach of direct laser pumping.

Looking further ahead, integrating multicolor solitons with active phase-locking could enable new approaches to optical frequency division and self-referencing. A decade after the first theoretical proposals, multicolor solitons have finally arrived on chip, and the coupled-ring platform that enabled them promises further surprises.
